# P108: Evaluation of hand hygiene products usability for health-care workers

**DOI:** 10.1186/2047-2994-2-S1-P108

**Published:** 2013-06-20

**Authors:** SY Baik, EK Kim, SK Hong

**Affiliations:** 1 Infection Control Office, CHA Bundang Medical Center, CHA university, Seongnam-si, Gyeonggi-do, Korea, Republic Of; 2 Department of Internal Medicine, CHA Bundang Medical Center, CHA university, Seongnam-si, Gyeonggi-do, Korea, Republic Of

## Introduction

Hand hygiene is known as the fundamental element for preventing health-care associated infection. The WHO recommends to select products in consider of user acceptance. We conducted a survey about hand hygiene products usability of health-care workers(HCWs) in order to figure out problems and utilize in next activity.

## Methods

On December 2009, A self-administered questionnaire survey on HCWs was carried out during 2 weeks in teaching hospital of 865 beds in Korea. The questionnaire was composed of 24 questions in 3 categories: 1) general characteristics 2) hand hygiene practice 3) hand hygiene product usability. The retrieved answers were analyzed by descriptive statistical analysis.

## Results

The survey was returned by 554 HCWs. The majority of participants were female(87.2%) and average age was 29.4(*SD*=6.15). In profession, participants were consisted of nurses(62.8%), technologists(17.5%), nursing assistants(14.6%) and physicians(4.2%). On the perception of practice, 65.7% of HCWs answered they had 10-30 occasions for hand hygiene per day and 44.6% of HCWs reported self-administered compliance by 50-80%. In usability, antimicrobial liquid soap(49.1%) was preferred to alcohol gel(31.6%). The most cleansed part was palm(62.3%) and uncleansed part was wrist(43.1%)and fingertip(29.8%). The reasons of non-compliance were “too busy”(63.5%), skin reaction(20.2%), lack of perception(19.9%), and resource shortage(17.5%). Many HCWs experienced skin irritation(52.7%). HCWs thought that “alcohol gel is convenient”(76.7%), “makes hands dry”(55.1%), “dry faster”(27.1%) and the antimicrobial liquid soap “makes hands dry”(51.6%), “foam insufficiently”(24.7%), “inconvenient”(14.1%).

## Conclusion

In this study, HCW’s self-administered performance is end up in middle level. Also, HCWs frequently misuse products, clean hands in inappropriate way and have various skin problems. It revealed needs for education about hand hygiene method, products usage and choice of products. We suggest further study about knowledge and perception among HCWs to improve hand hygiene compliance.

## Disclosure of interest

None declared

